# Mirabegron for the treatment of overactive bladder: a prespecified pooled efficacy analysis and pooled safety analysis of three randomised, double-blind, placebo-controlled, phase III studies

**DOI:** 10.1111/ijcp.12194

**Published:** 2013-05-21

**Authors:** V W Nitti, V Khullar, P Kerrebroeck, S Herschorn, J Cambronero, J C Angulo, M B Blauwet, C Dorrepaal, E Siddiqui, N E Martin

**Affiliations:** 1 Department of Urology, NYU Langone Medical CenterNew York, NY, USA; 2 Urogynaecology Department, St Mary's Hospital, Imperial CollegeLondon, UK; 3 Department of Urology, Maastricht University Medical CenterMaastricht, the Netherlands; 4 Division of Urology, University of TorontoToronto, ON, Canada; 5 Department of Urology, Infanta Leonor HospitalMadrid, Spain; 6 Department of Urology, Hospital Universitario De GetafeMadrid, Spain; 7 Astellas Pharma Global Development, Inc., Global Data Science – BiostatisticsNorthbrook, IL, USA; 8 Astellas Pharma Global Development – EU, Global Medical Science – UrologyLeiderdorp, the Netherlands; 9 Astellas Pharma Europe LtdSurrey, UK; 10 Department of Urology, Ealing HospitalLondon, UK; 11 Astellas Scientific and Medical Affairs IncNorthbrook, IL, USA

## Abstract

**Introduction:**

To examine pooled efficacy data from three, large phase III studies comparing mirabegron (50 and 100 mg) with placebo, and pooled safety data including additional mirabegron 25 mg and tolterodine extended release (ER) 4 mg results.

**Methods:**

This prespecified pooled analysis of three randomised, double-blind, placebo-controlled, 12-week studies, evaluated efficacy and safety of once-daily mirabegron 25 mg (safety analysis), 50 or 100 mg (efficacy and safety analyses) and tolterodine ER 4 mg (safety analysis) for the treatment of symptoms of overactive bladder (OAB). Co-primary efficacy measures were change from baseline to Final Visit in the mean number of incontinence episodes/24 h and mean number of micturitions/24 h. Key secondary efficacy end-points included mean number of urgency episodes/24 h and mean volume voided/micturitions, while other end-points included patient-reported outcomes according to the Treatment Satisfaction-Visual Analogue Scale (TS-VAS) and responder analyses [dry rate (posttreatment), ≥ 50% reduction in incontinence episodes/24 h, ≤ 8 micturitions/24 h (*post hoc* analysis)]. The safety analysis included adverse event (AE) reporting, laboratory assessments, ECG, postvoid residual volume and vital signs (blood pressure, pulse rate).

**Results:**

Mirabegron (50 and 100 mg once daily) demonstrated statistically significant improvements compared with placebo for the co-primary end-points, key secondary efficacy variables, TS-VAS and responder analyses (all comparisons p < 0.05). Mirabegron is well tolerated and demonstrates a good safety profile. The most common AEs (≥ 3%) included hypertension, nasopharyngitis and urinary tract infection (UTI); the incidence of hypertensive events and UTIs decreased with increasing dose. For mirabegron, the incidence of the bothersome antimuscarinic AE, dry mouth, was at placebo level and of a lesser magnitude than tolterodine.

**Conclusion:**

The efficacy and safety of mirabegron are demonstrated in this large pooled clinical trial dataset in patients with OAB.

**What's known**

- Overactive bladder (OAB) is a chronic condition that is increasingly prevalent with age and represents an increasing healthcare burden to society.
- Antimuscarinic drugs are the current mainstay of pharmacologic treatment for OAB, but are commonly associated with adherence-limiting adverse effects including dry mouth, constipation and blurred vision.
- Many patients discontinue antimuscarinic therapy or seek alternative approaches to control OAB as a result of adverse effects or suboptimal efficacy.

**What's new**

- Mirabegron is a β_3_-adrenoceptor agonist, the first in a new class of drugs with a mode of action that is different from antimuscarinic agents.
- Mirabegron stimulates the β_3_-adrenoceptors in the bladder leading to detrusor relaxation and increased bladder capacity.
- In three international, multicentre, phase III studies, comparing mirabegron, at doses of 25, 50 and 100 mg, with placebo, mirabegron was associated with significant improvements in incontinence episodes and micturition frequency, and was well tolerated.

## Introduction

Overactive bladder (OAB) is defined by the International Continence Society as urinary urgency, with or without urinary incontinence, usually with frequency and nocturia, with no proven infection or other obvious pathology 1,2. Lower urinary tract symptoms, including those associated with OAB, are prevalent in approximately half of the general population 3, are often debilitating and have a significant impact on patients’ daily routines and quality of life 3–5. OAB affects 12–16% of the adult population across Europe, the USA and Japan 6–8, and its prevalence increases with age, affecting approximately 30% of individuals > 65 years of age 9, therefore representing a substantive health problem for the ageing population.

Normal bladder function is regulated by the synergistic actions of the autonomic nervous system: parasympathetic nerves stimulate contraction of the detrusor muscle via muscarinic receptors while stimulation of the sympathetic nerves leads to detrusor relaxation via β-adrenoceptors. The central pathophysiology of OAB involves involuntary contractions of the detrusor muscle as a result of increased afferent activity, decreased inhibitory control from the central nervous system, and/or increased sensitivity of the detrusor muscle to parasympathetic stimulation 10,11.

Because of the role of β-adrenoceptors in relaxation of the detrusor, research was aimed at identifying an effective and well-tolerated agent directed at the β-adrenoceptor. The β_3_-adrenoceptor was recognised as the predominant β-receptor subtype in the human urinary bladder, representing 97% of total β-adrenoceptor mRNA expression in the human bladder 12. The importance of its role in the relaxation of the detrusor has been confirmed by *in vitro* pharmacological studies using human bladder strips 13–15. In animal models, activation of the β-adrenoceptor in the bladder facilitates urine storage through flattening and lengthening of the bladder base 16. Furthermore, stimulation of the β_3_-adrenoceptor is associated with increased bladder capacity, without change in micturition pressure, residual volume or voiding contraction 17–19. As a consequence, targeting the β_3_-adrenoceptor increases the storage capacity of the bladder without affecting the amplitude of the voiding contraction.

Mirabegron, a β_3_-adrenoceptor agonist approved in Japan, the USA, Canada and Europe, for the treatment of OAB symptoms, is the first of a new class of compounds with a mechanism of action that is different from antimuscarinic agents 20. The recommended starting dose of mirabegron is 25 mg in the USA, which can be increased to 50 mg based on individual efficacy and tolerability, and 50 mg in Japan and Europe; the 100 mg dose is not approved for use.

Three large-scale, 12-week, phase III studies conducted in the USA/Canada (study 047; NCT00662909) 21; Europe and Australia (study 046; NCT00689104) 22; and Europe/USA/Canada (study 074; NCT00912964)^23^ have demonstrated the safety, tolerability and superior efficacy of mirabegron at doses of 25 mg (NCT00912964 only), 50 mg (all three studies) and 100 mg (NCT00662909 and NCT00689104) compared with placebo for the treatment of OAB symptoms. The pooled efficacy analysis was prespecified and excluded the 25 mg mirabegron dose because it was limited to a single study. The safety analysis was an aggregate of all data from the three phase III studies and included the 25, 50 and 100 mg mirabegron doses. Pooling these data allows for additional analyses of efficacy and safety in a large dataset representative of the general OAB population in terms of demographics, previous OAB history and prevalence of comorbidities. In particular, it is important to examine the efficacy, safety and tolerability profile demonstrated in the individual studies in the pooled population for overall effect size as well as safety data reporting across the treatment groups. These pooled mirabegron data evaluations inform benefit–risk assessments and support judicious dose selection.

## Methods

### Study design

**Figure 1:**
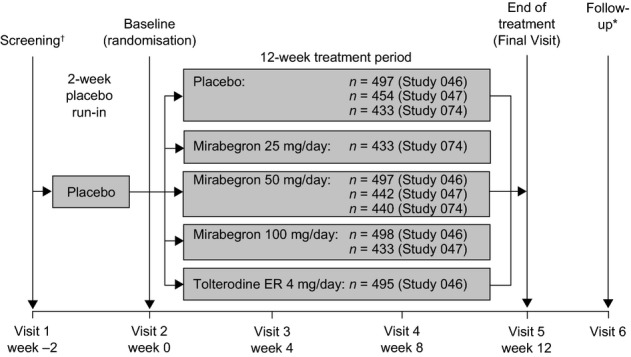
Overview of the individual phase III studies included in the pooled analyses. ^†^Screening from weeks −3 to −2; *Evaluation of adverse events and concomitant medication by telephone contact or visit 30 days after Final Visit in studies 046 and 047 and 2 weeks after Final Visit in Study 074. *n,* number of patients randomised

This was a prespecified pooled analysis of three randomised, double-blind, placebo-controlled, 12-week studies, which evaluated the efficacy of mirabegron 50 and 100 mg, and safety of mirabegron 25 mg (NCT00912964 only), 50 mg or 100 mg for the treatment of symptoms of OAB. Each study shared a similar design, although the assessed doses of mirabegron and inclusion of an active control arm with tolterodine extended release (ER) 4 mg varied across trials (Figure 1). Male and female patients aged ≥ 18 years with OAB symptoms for ≥ 3 months were enrolled. Following a 2-week, single-blind, placebo run-in period to determine baseline symptoms and patient eligibility, patients were randomised if, during a 3-day micturition diary period, they recorded ≥ 8 micturitions/24 h and ≥ 3 urgency episodes – based on urgency grade of 3 or 4 according to the Patient Perception of Intensity and Urgency Scale [PPIUS 24] – with or without urgency incontinence. Patients were excluded with stress incontinence or mixed incontinence with stress predominance at screening or had an average total daily urine volume > 3000 ml.

Patients were randomised to receive once-daily oral placebo, mirabegron 25 mg (NCT00912964 only), mirabegron 50 mg (NCT00912964/NCT00689104/NCT00662909), mirabegron 100 mg (NCT00689104/NCT00662909) or tolterodine ER 4 mg (NCT00689104 only) for 12 weeks using a computer-generated randomisation scheme; after assignment to treatment, both patient and study investigator were blinded to the identity of the study drug. Efficacy data for treatment arms that were represented in only one study (i.e. mirabegron 25 mg or tolterodine ER 4 mg) were not pooled as these data were only available from one trial. In summary, the efficacy data in these pooled analyses relate to the placebo and 50 mg mirabegron groups from all three phase III studies, and the 100 mg mirabegron group from two of the studies [NCT00662909 21/NCT00689104 22]. In contrast, the pooled 12-week phase III safety analysis includes data for the placebo, 25, 50 and 100 mg mirabegron doses and the tolterodine ER 4 mg active control arm.

### Efficacy end-points

Efficacy measures were recorded in a patient micturition diary over a 3-day period prior to clinic visits which occurred at baseline and 4, 8 and 12 weeks/Final Visit (end of treatment, i.e. last on-treatment assessment including patients who did not complete week 12 visit) after randomisation to treatment. The co-primary end-points in all three studies were change from baseline to Final Visit in the mean number of incontinence episodes/24 h and mean number of micturitions/24 h. Key secondary efficacy end-points in all three studies were change from baseline to Final Visit in the mean volume voided/micturition, change from baseline to week 4 in the mean number of incontinence episodes/24 h, and change from baseline to week 4 in the mean number of micturitions/24 h. Study 074 (NCT00912964) had three additional key secondary end-points – mean level of urgency (based on the PPIUS), mean number of urgency episodes (PPIUS grade 3 or 4)/24 h, and the mean number of urgency incontinence episodes/24 h – which were also included as additional secondary end-points in Study 046 (NCT00689104) and Study 047 (NCT00662909) but did not undergo the hierarchical testing procedure for multiple end-points. Further secondary end-points included change from baseline to Final Visit in mean number of nocturia episodes/24 h and patients considered ‘responders’ to treatment, i.e. the proportion of patients with incontinence episode(s) at baseline with zero incontinence at Final Visit, the proportion of patients with a ≥ 50% decrease from baseline in mean number of incontinence episodes/24 h, and a *post hoc* analysis of the proportion of patients with ≤ 8 micturitions/24 h at Final Visit. Patient-reported outcomes were assessed using the Treatment Satisfaction-Visual Analogue Scale (TS-VAS), a quantitative instrument measuring subjective improvement in patients with OAB. Complete satisfaction was indicated by a score of 10, with positive change from baseline indicating improvement.

### Safety end-points

The analysis of the pooled safety data included adverse event (AE) reporting, laboratory parameters (haematology and serum chemistry), vital signs (pulse rate and blood pressure), ECG data and postvoid residual (PVR) volume. AEs of interest were based on potential or theoretical risk related to the pharmacology of β_3_-agonists, observed findings in non-clinical or clinical data, or recommended monitoring for specific events. The most common treatment-emergent AEs (TEAEs), according to the preferred term, are reported herein as TEAEs in ≥ 3% of the total mirabegron population. Hypertension was reported as an AE if average systolic blood pressure (SBP) was ≥ 140 mmHg and/or average diastolic blood pressure (DBP) ≥ 90 mmHg at two consecutive postbaseline visits for normotensive patients at baseline; average SBP increased ≥ 20 mmHg and/or average DBP increased ≥ 10 mmHg at two consecutive postbaseline visits for patients with hypertension at baseline; initiation or increase in antihypertensive medication; or according to clinical assessment by the investigator. Tachycardia was to be reported as an AE if average AM or PM resting pulse rate measured by the patient over 3 days was > 100 bpm (unless in the investigator's opinion this did not constitute an AE of tachycardia). Urinary retention was summarised as a reported TEAE based on spontaneous reporting by the investigator, without a prespecified definition per protocol, and assessed concurrently with changes in PVR volume (change from baseline of ≥ 150 ml).

### Statistical analyses

The safety analysis set (SAF) comprised all randomised patients who took ≥ 1 dose of double-blind study drug; the full analysis set (FAS) comprised SAF patients who had ≥ 1 micturition measurement at baseline and ≥ 1 postbaseline micturition measurement; the FAS-incontinence (FAS-I) set comprised FAS patients who reported ≥ 1 incontinence episode at baseline. Efficacy analyses were performed using the FAS except for incontinence episode end-points which used the FAS-I. Safety analyses were performed using the SAF. Descriptive statistics were used for baseline demographic and clinical characteristics.

For the efficacy analysis, data for the placebo and 50 mg mirabegron groups from all three studies were pooled, and data for the 100 mg mirabegron groups from two studies were pooled. Hypothesis testing on the change from baseline in incontinence episodes was performed using a separate stratified rank analysis of covariance (ANCOVA) for each pairwise treatment group difference (mirabegron 50 and 100 mg vs. placebo). For all other efficacy end-points, hypothesis testing comparing mirabegron 50 and 100 mg vs. placebo on the change from baseline values was performed using an ANCOVA model including treatment, gender and study as fixed factors, and baseline value as a covariate. Based on the ANCOVA, least squares mean estimates and two-sided 95% confidence intervals (CIs) for mean changes from baseline were derived within treatment groups and between each mirabegron treatment group and placebo on all efficacy end-points. Multiplicity between the two primary and six key secondary efficacy end-points was controlled at the α = 0.05 type I error rate using a stepwise parallel gatekeeping procedure in which the placebo-corrected difference in mean change from baseline for each mirabegron dose had to be statistically significant to allow hypothesis testing to proceed to the next end-point step. The Hochberg procedure was performed at the α = 0.05 significance level for comparisons of the 50 and 100 mg mirabegron treatment groups with placebo to adjust for multiplicity within each stage. If only one mirabegron dose group proceeded to the next stage for any efficacy end-point, the comparison with placebo was assessed at the α = 0.025 level. Responder end-points were analysed using a logistic regression model which included treatment, gender, study and baseline value.

For the pooled safety analysis, data from all three studies were included: placebo, 25, 50 and 100 mg mirabegron groups, and tolterodine ER 4 mg arm. All AEs were coded using the Medical Dictionary for Regulatory Activities version 12.1. TEAEs were defined as any AE which started, or worsened, after the first dose of study drug through 30 days after the last dose of study drug. Drug-related TEAEs included those AEs reported as probably or possibly related to study drug as assessed by the investigator or if the AE had a missing relationship. Descriptive statistics were used for measurements of laboratory parameters, ECGs and PVR volume. Change from baseline values for vital sign variables was analysed using the ANCOVA model described above.

## Results

### Demographic and baseline clinical characteristics

**Figure 2:**
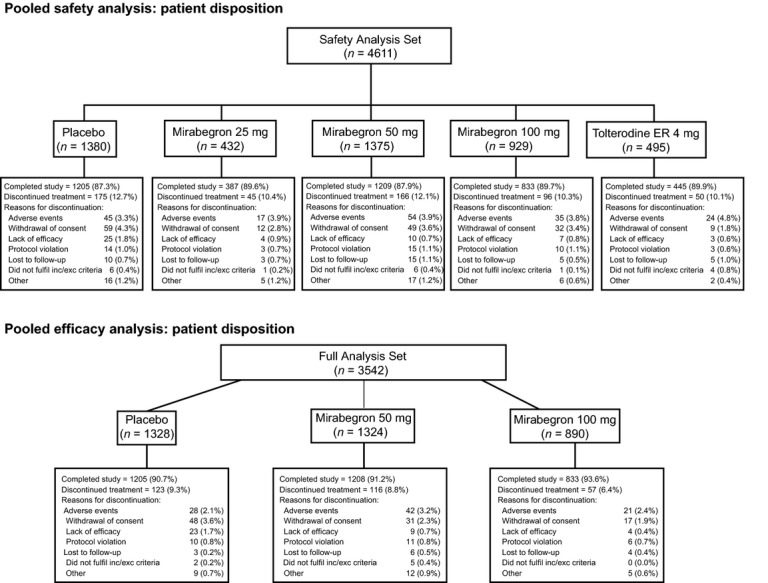
Patient disposition in the pooled efficacy and safety analyses

Overall, there were a total of 3542 patients (placebo, *n* = 1328; mirabegron 50 mg, *n* = 1324; mirabegron 100 mg, *n* = 890) and 2317 patients (placebo, *n* = 878; mirabegron 50 mg, *n* = 862; mirabegron 100 mg, *n* = 577), respectively, in the FAS and FAS-I population for the pooled efficacy analysis. The pooled safety analysis included the additional 25 mg mirabegron dose and tolterodine active control arm and consisted of 4611 patients (mirabegron 25 mg, *n* = 432; mirabegron 50 mg, *n* = 1375; mirabegron 100 mg, *n* = 929; placebo, *n* = 1380; tolterodine, *n* = 495) who took at least one dose of double-blind study medication. In the FAS and SAF analyses, the disposition and reasons for discontinuation of study drug were similar for all treatment groups (Figure 2).

Demographic and baseline characteristics were similar between the FAS and FAS-I population with the exception of gender; approximately 72% and 82% of patients were female in the FAS and FAS-I population, respectively (Table 1). Of the patients who had received prior antimuscarinic OAB medication, approximately two-thirds discontinued prior OAB treatment because of insufficient efficacy, and approximately one-quarter because of poor tolerability. The proportion discontinuing prior OAB treatment for either reason was similar across treatment groups (Table 1).

**Table 1:** Patient demographics and baseline clinical characteristics by treatment group (FAS)

	Placebo (*n* = 1328)	Mirabegron 50 mg (*n* = 1324)	Mirabegron 100 mg (*n* = 890)
**Gender, *n* (%)**			
Male	362 (27.3)	382 (28.9)	241 (27.1)
Female	966 (72.7)	942 (71.1)	649 (72.9)
**Age (years)**			
Mean (SD)	59.2 (13.2)	59.7 (12.6)	59.8 (12.9)
Range	20–95	21–91	19–90
**Race, *n* (%)**			
White	1227 (92.4)	1235 (93.3)	838 (94.2)
Black or African American	80 (6.0)	61 (4.6)	36 (4.0)
Asian	13 (1.0)	17 (1.3)	8 (0.9)
Other	8 (0.6)	11 (0.8)	8 (0.9)
BMI (kg/m^2^)*			
Mean	29.1 (6.3)	29.0 (6.1)	29.1 (6.1)
Range	15.9–58.1	16.3–60.5	16.2–62.6
**Type of OAB, *n* (%)**			
Urgency incontinence	442 (33.3)	491 (37.1)	297 (33.4)
Mixed	415 (31.3)	412 (31.1)	271 (30.4)
Frequency	471 (35.5)	421 (31.8)	322 (36.2)
**Duration of OAB (months)**			
Mean (SD)	86.3 (99.1)	85.2 (93.1)	88.3 (101.6)
**Previous OAB drug, *n* (%)**			
Yes	704 (53.0)	688 (52.0)	460 (51.7)
**Reasons for previous OAB drug discontinuation, *n* (%)**†			
Insufficient effect			
Yes	466 (66.2)	464 (67.4)	296 (64.3)
No	238 (33.8)	224 (32.6)	164 (35.7)
Poor tolerability			
Yes	185 (26.3)	173 (25.1)	113 (24.6)
No	519 (73.7)	515 (74.9)	347 (75.4)
**Mean number of incontinence episodes/24 h**			
Mean (SD)	1.8 (2.5)	1.8 (2.5)	1.8 (2.4)
Range	0–26	0–18	0–15
**Mean number of micturitions/24 h**			
Mean (SD)	11.6 (3.1)	11.7 (3.2)	11.6 (3.0)
Range	4–40	6–37	7–35

FAS, full analysis set; SD, standard deviation; BMI, body mass index; OAB, overactive bladder.

* For BMI, placebo (*n* = 1327) and mirabegron 100 mg (*n* = 889).

† Percentages are based on patients who had taken previous OAB medication. Patients could choose more than one reason for discontinuation.

### Efficacy outcomes

**Figure 3:**
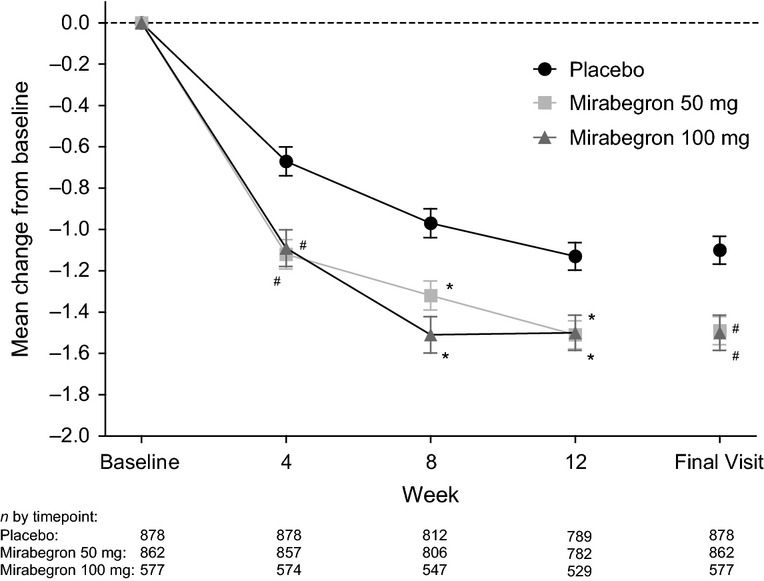
Adjusted mean change from baseline (±SE) by time point in the mean number of incontinence episodes/24 h for the pooled placebo, mirabegron 50 and 100 mg groups (FAS-I). ^#^Statistically significant treatment benefit relative to placebo (p < 0.05) with multiplicity adjustment. *Statistically significant treatment benefit relative to placebo (p < 0.05) without multiplicity adjustment. SE, standard error; FAS-I, full analysis set-incontinence.

**Figure 4:**
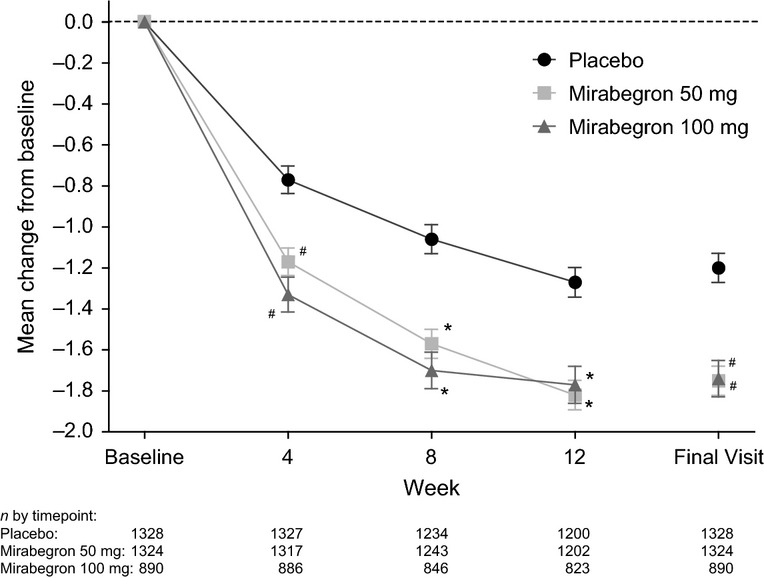
Adjusted mean change from baseline (±SE) by time point in mean number of micturitions/24 h for the pooled placebo, mirabegron 50 and 100 mg groups (FAS). ^#^Statistically significant treatment benefit relative to placebo (p < 0.05) with multiplicity adjustment. *Statistically significant treatment benefit relative to placebo (p < 0.05) without multiplicity adjustment. SE, standard error; FAS, full analysis set.

Mirabegron at doses of 50 and 100 mg once daily demonstrated statistically significant improvement from baseline to Final Visit compared with placebo in reducing the mean number of incontinence episodes/24 h and the mean number of micturitions/24 h (p < 0.05 for all comparisons; Table 2 and Figures 3 and 4, respectively). The adjusted mean (95% CI) change from baseline to Final Visit in the mean number of incontinence episodes/24 h was −1.10 (−1.23, −0.97), −1.49 (−1.63, −1.36) and −1.50 (−1.67, −1.34) in the placebo, mirabegron 50 and 100 mg groups, respectively, and −1.20 (−1.34, −1.06), −1.75 (−1.89, −1.61), −1.74 (−1.91, −1.56) for the adjusted mean change from baseline to Final Visit in the mean number of micturitions/24.

**Table 2:** Summary of primary and secondary efficacy end-points (FAS and FAS-I)

	Placebo	Mirabegron 50 mg	Mirabegron 100 mg
**Co-primary end-points**			
Change from baseline to Final Visit in the mean number of incontinence episodes/24 h (FAS-I)			
Baseline	2.73 (0.09)	2.71 (0.09)	2.79 (0.10)
Final Visit	1.64 (0.09)	1.23 (0.08)	1.25 (0.09)
Change from baseline	−1.09 (0.09)	−1.48 (0.08)	−1.54 (0.09)
Adjusted change from baseline* (95% CI)	−1.10 (−1.23, −0.97)	−1.49 (−1.63, −1.36)	−1.50 (−1.67, −1.34)
Adjusted difference vs. placebo* (95% CI)	–	−0.40 (−0.58, −0.21)3	−0.41 (−0.62, −0.19)3
Change from baseline to Final Visit in the mean number of micturitions/24 h (FAS)			
Baseline	11.58 (0.09)	11.70 (0.09)	11.58 (0.10)
Final Visit	10.39 (0.09)	9.93 (0.09)	9.83 (0.11)
Change from baseline	−1.18 (0.08)	−1.77 (0.08)	−1.75 (0.09)
Adjusted change from baseline* (95% CI)	−1.20 (−1.34, −1.06)	−1.75 (−1.89, −1.61)	−1.74 (−1.91, −1.56)
Adjusted difference vs. placebo* (95% CI)	–	−0.55 (−0.75, −0.36)†	−0.54 (−0.77, −0.31)†
**Key secondary end-points**			
Change from baseline to Final Visit in the mean volume voided/micturition (FAS)			
Baseline	159.2 (1.54)	159.0 (1.55)	157.9 (1.89)
Final Visit	168.6 (1.90)	180.2 (2.01)	179.9 (2.39)
Change from baseline	9.4 (1.31)	21.2 (1.31)	22.0 (1.52)
Adjusted change from baseline* (95% CI)	9.4 (6.9, 12.0)	21.4 (18.8, 23.9)	21.7 (18.5, 24.9)
Adjusted difference vs. placebo* (95% CI)	–	11.9 (8.3, 15.5)†	12.3 (8.1, 16.5)†
Change from baseline to week 4 in the mean number of incontinence episodes/24 h (FAS-I)			
Baseline	2.73 (0.09)	2.71 (0.09)	2.79 (0.10)
Week 4	2.06 (0.10)	1.59 (0.08)	1.69 (0.10)
Change from baseline	−0.67 (0.08)	−1.12 (0.08)	−1.10 (0.09)
Adjusted change from baseline* (95% CI)	−0.67 (−0.81, −0.54)	−1.12 (−1.26, −0.98)	−1.09 (−1.27, −0.92)
Adjusted difference vs. placebo* (95% CI)	–	−0.45 (−0.64, −0.26)†	−0.42 (−0.65, −0.20)†
Change from baseline to week 4 in the mean number of micturitions/24 h (FAS)			
Baseline	11.58 (0.09)	11.71 (0.09)	11.58 (0.10)
Week 4	10.82 (0.09)	10.52 (0.10)	10.26 (0.11)
Change from baseline	−0.76 (0.07)	−1.19 (0.07)	−1.32 (0.09)
Adjusted change from baseline* (95% CI)	−0.77 (−0.90, −0.64)	−1.17 (−1.30, −1.04)	−1.33 (−1.50, −1.16)
Adjusted difference vs. placebo* (95% CI)	–	−0.40 (−0.59, −0.22)†	−0.56 (−0.78, −0.35)†
Mean level of urgency (FAS)			
Baseline	2.39 (0.02)	2.42 (0.02)	2.46 (0.02)
Final Visit	2.25 (0.02)	2.15 (0.02)	2.19 (0.02)
Change from baseline	−0.14 (0.02)	−0.27 (0.02)	−0.27 (0.02)
Adjusted change from baseline* (95% CI)	−0.15 (−0.18, −0.12)	−0.26 (−0.30, −0.23)	−0.26 (−0.30, −0.22)
Adjusted difference vs. placebo* (95% CI)	–	−0.11 (−0.16, −0.07)†	−0.11 (−0.16, −0.06)†
Mean number of urgency incontinence episodes/24 h (FAS−I)			
Baseline	2.42 (0.08)	2.42 (0.08)	2.53 (0.10)
Final Visit	1.46 (0.08)	1.05 (0.07)	1.11 (0.09)
Change from baseline	−0.96 (0.08)	−1.37 (0.07)	−1.42 (0.09)
Adjusted change from baseline* (95% CI)	−0.98 (−1.10, −0.86)	−1.38 (−1.50, −1.26)	−1.38 (−1.53, −1.23)
Adjusted difference vs. placebo* (95% CI)	–	−0.40 (−0.57, −0.23)†	−0.40 (−0.60, −0.20)†
Mean number of urgency episodes (PPIUS grade 3 or 4)/24 h (FAS)			
Baseline	5.61 (0.10)	5.80 (0.10)	5.96 (0.12)
Final Visit	4.38 (0.11)	3.85 (0.11)	4.02 (0.13)
Change from baseline	−1.23 (0.10)	−1.95 (0.10)	−1.94 (0.12)
Adjusted change from baseline* (95% CI)	−1.29 (−1.47, −1.11)	−1.93 (−2.11, −1.75)	−1.89 (−2.11, −1.66)
Adjusted difference vs. placebo* (95% CI)	–	−0.64 (−0.89, −0.39)†	−0.60 (−0.89, −0.31)†
**Additional secondary end-points**			
Change from baseline to Final Visit in mean number of nocturia episodes/24 h (FAS)			
Baseline	2.18 (0.04)	2.22 (0.04)	2.25 (0.05)
Final Visit	1.78 (0.04)	1.66 (0.04)	1.71 (0.05)
Change from baseline	−0.41 (0.04)	−0.56 (0.04)	−0.54 (0.04)
Adjusted change from baseline* (95% CI)	−0.42 (−0.48, −0.35)	−0.55 (−0.62, −0.49)	−0.54 (−0.62, −0.46)
Adjusted difference vs. placebo* (95% CI)	–	−0.14 (−0.23, −0.05)‡	−0.12 (−0.23, −0.02)‡
Change from baseline to Final Visit in TS-VAS (FAS)			
Baseline	4.87 (0.11)	4.82 (0.11)	4.43 (0.13)
Final Visit	6.05 (0.10)	6.79 (0.09)	6.94 (0.11)
Change from baseline	1.18 (0.11)	1.96 (0.12)	2.51 (0.14)
Adjusted change from baseline* (95% CI)	1.25 (1.08, 1.42)	2.01 (1.84, 2.19)	2.33 (2.11, 2.55)
Adjusted difference vs. placebo* (95% CI)	–	0.76 (0.52, 1.01)‡	1.08 (0.80, 1.37)‡

All values mean (SE) unless otherwise stated.

* Estimates are based on an analysis of covariance model, which included treatment group, gender and study as fixed factors, and baseline as a covariate.

† p < 0.05 vs. placebo with multiplicity adjustment.

‡ p < 0.05 vs. placebo without multiplicity adjustment.

The key secondary end-points, change from baseline to week 4 (first time point assessed) in the mean number of incontinence episodes/24 h and mean number of micturitions/24 h, were significantly improved for both mirabegron doses compared with placebo (p < 0.05, all comparisons; Table 2 and Figures 3 and 4, respectively). The adjusted mean (95% CI) change from baseline to week 4 in the mean number of incontinence episodes/24 h was −0.67 (−0.81, −0.54), −1.12 (−1.26, −0.98) and −1.09 (−1.27, −0.92) in the placebo, mirabegron 50 and 100 mg groups, respectively. The adjusted mean (95% CI) change from baseline to week 4 in the mean number of micturitions/24 h was −0.77 (−0.90, −0.64), −1.17 (−1.30, −1.04) and −1.33 (−1.50, −1.16) in the placebo, mirabegron 50 and 100 mg groups, respectively.

**Figure 5:**
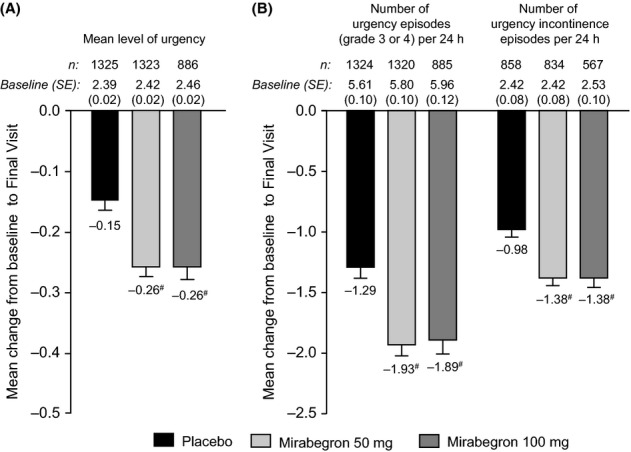
Adjusted mean change from baseline to Final Visit (SE) in key secondary end-points: (A) mean level of urgency (FAS) (B) number of urgency episodes/24 h (FAS) and number of urgency incontinence episodes/24 h (FAS-I). ^#^Statistically significant treatment benefit compared with placebo (p < 0.05) with multiplicity adjustment. SE, standard error; FAS, full analysis set; FAS-I, full analysis set-incontinence.

Both 50 and 100 mg mirabegron doses were associated with statistically significant improvements from baseline to Final Visit compared with placebo for the key secondary efficacy end-points of mean volume voided/micturition (p < 0.05 for all comparisons; Table 2), mean level of urgency, mean number of urgency episodes (grade 3 or 4)/24 h and mean number of urgency incontinence episodes/24 h (p < 0.05 for all comparisons; Table 2 and Figure 5).

Significant improvements were also evident in the pooled efficacy analysis with respect to the change from baseline to Final Visit (95% CI) in the mean number of nocturia episodes/24 h in the mirabegron 50 mg [−0.55 (−0.62, −0.49)] and mirabegron 100 mg groups [−0.54 (−0.62, −0.46)] vs. placebo [−0.42 (−0.48, −0.35)] (p < 0.05; Table 2).

For the responder analyses, the percentage of patients with zero incontinence episodes at Final Visit (‘dry rate’) was significantly higher in the mirabegron 50 and 100 mg groups (44.1% and 46.4%, respectively) compared with placebo (37.8%). The corresponding odds ratios (95% CI) for the mirabegron 50 and 100 mg groups were 1.32 (1.08, 1.61) and 1.58 (1.25, 2.00), respectively (p < 0.05). The percentage of patients with a ≥ 50% reduction from baseline to Final Visit in mean number of incontinence episodes/24 h was also significantly higher in the mirabegron 50 and 100 mg groups (69.5% and 70.5%, respectively) compared with placebo (59.6%). The corresponding odds ratios (95% CI) for the mirabegron 50 and 100 mg groups were 1.54 (1.26, 1.89) and 1.64 (1.29, 2.07), respectively (p < 0.05). In the *post hoc* responder analysis, both 50 and 100 mg mirabegron doses were also associated with a significant improvement in ‘micturition normalisation’ (i.e. the percentage of patients with ≤ 8 micturitions/24 h) at Final Visit (31.6% and 34.0%) compared with placebo (24.6%). The corresponding odds ratios (95% CI) for the mirabegron 50 and 100 mg groups were 1.57 (1.30, 1.89) and 1.69 (1.37, 2.09), respectively (p < 0.05).

In health-related quality of life outcomes, mirabegron 50 and 100 mg was associated with a statistically significant improvement in the TS-VAS score at Final Visit compared with placebo (p < 0.05; Table 2). The adjusted mean (95% CI) changes from baseline to Final Visit were 1.25 (1.08, 1.42), 2.01 (1.84, 2.19) and 2.33 (2.11, 2.55) for the placebo, mirabegron 50 and 100 mg groups, respectively.

### Efficacy in patients with prior antimuscarinic use and treatment-naïve patients

Both mirabegron 50 and 100 mg doses improved the co-primary end-points in patients who had discontinued previous antimuscarinic medication and in treatment-naïve patients. The adjusted mean difference (95% CI) from baseline to Final Visit in the mean number of incontinence episodes/24 h in patients who had discontinued previous antimuscarinic medication was −0.92 (−1.09, −0.75), −1.49 (−1.66, −1.32) and −1.42 (−.64, −1.21) in the placebo, mirabegron 50 mg and mirabegron 100 mg groups, respectively, and −1.35 (−1.55, −1.14), −1.50 (−1.71, −1.29) and −1.62 (−1.87, −1.36) in treatment-naïve patients.

The adjusted mean difference (95% CI) in the mean number of micturitions/24 h from baseline to Final Visit in the placebo, mirabegron 50 and 100 mg groups, respectively, was −0.93 (−1.12, −0.74), −1.67 (−1.86, −1.48) and −1.61 (−1.85, −1.37) in patients who discontinued previous antimuscarinic therapy, and −1.51 (−1.71, −1.31), −1.84 (−2.04, −1.64) and −1.87 (−2.12, −1.63) in treatment-naïve patients. The adjusted mean difference vs. placebo in patients who discontinued previous antimuscarinic therapy was of a greater magnitude than in treatment-naïve patients because of the higher placebo response in treatment-naïve patients.

### Safety outcomes

The overall incidence of TEAEs was similar across treatment groups (Table 3) and there was no evidence of a dose–response relationship across the mirabegron treatment groups for overall rates of TEAEs: placebo (47.7%), mirabegron 25 mg (48.6%), mirabegron 50 mg (47.1%), mirabegron 100 mg (43.3%), total mirabegron (46.0%) and tolterodine ER 4 mg (46.7%). The most common TEAEs reported in ≥ 3% of the total mirabegron group were hypertension, nasopharyngitis and urinary tract infection (Table 3). The incidence of hypertension and urinary tract infection decreased as the dose of mirabegron increased from 25 to 100 mg. The most common drug-related TEAEs in the mirabegron groups were hypertension and headache, which were of similar incidence with placebo and tolterodine, with the exception of dry mouth, which was at placebo level for mirabegron but of a higher magnitude in the tolterodine group (Table 3). The majority of AEs were of mild or moderate severity across treatment groups.

**Table 3:** Overview of treatment-emergent adverse events in the pooled safety analysis (SAF)

		Mirabegron				
			
Number of patients (%)	Placebo (*n* = 1380)	25 mg (*n* = 432)	50 mg (*n* = 1375)	100 mg (*n* = 929)	Total (*n* = 2736)	Tolterodine ER 4 mg (*n* = 495)
Any TEAE	658 (47.7)	210 (48.6)	647 (47.1)	402 (43.3)	1259 (46.0)	231 (46.7)
Drug-related TEAE	232 (16.8)	87 (20.1)	256 (18.6)	172 (18.5)	515 (18.8)	131 (26.5)
TEAE leading to discontinuation	46 (3.3)	17 (3.9)	53 (3.9)	34 (3.7)	104 (3.8)	22 (4.4)
Drug-related TEAE leading to discontinuation	27 (2.0)	11 (2.5)	35 (2.5)	25 (2.7)	71 (2.6)	20 (4.0)
SAE	29 (2.1)	7 (1.6)	29 (2.1)	26 (2.8)	62 (2.3)	11 (2.2)
Drug-related SAE	6 (0.4)	3 (0.7)	7 (0.5)	3 (0.3)	13 (0.5)	6 (1.2)
***Common TEAEs by preferred term (reported by ≥ 3% in total mirabegron group)***						
Hypertension	105 (7.6)	49 (11.3)	103 (7.5)	48 (5.2)	200 (7.3)	40 (8.1)
Nasopharyngitis	35 (2.5)	15 (3.5)	54 (3.9)	25 (2.7)	94 (3.4)	14 (2.8)
Urinary tract infection	25 (1.8)	18 (4.2)	40 (2.9)	25 (2.7)	83 (3.0)	10 (2.0)
***Antimuscarinic AEs of interest by preferred term (reported by ≥ 2% in any group)***						
Headache	43 (3.1)	10 (2.3)	47 (3.4)	23 (2.5)	80 (2.9)	18 (3.6)
Dry mouth	29 (2.1)	8 (1.9)	23 (1.7)	23 (2.5)	54 (2.0)	50 (10.1)
Constipation	20 (1.4)	7 (1.6)	22 (1.6)	15 (1.6)	44 (1.6)	10 (2.0)
Drug-related* ***TEAEs by preferred term (reported by ≥ 2% in any group)***						
Hypertension	63 (4.6)	30 (6.9)	65 (4.7)	32 (3.4)	127 (4.6)	30 (6.1)
Headache	18 (1.3)	4 (0.9)	28 (2.0)	12 (1.3)	44 (1.6)	11 (2.2)
Dry mouth	22 (1.6)	7 (1.6)	13 (0.9)	20 (2.2)	40 (1.5)	47 (9.5)

SAF, safety analysis set; ER, extended release; TEAE, treatment-emergent adverse event; SAE, serious adverse event.

* Possible or probable, as assessed by the investigator, or records where relationship was missing.

The proportion of patients discontinuing study drug because of TEAEs was low and similar across each group: placebo (3.3%), mirabegron 25 mg (3.9%), mirabegron 50 mg (3.9%), mirabegron 100 mg (3.7%), total mirabegron (3.8%) and tolterodine ER 4 mg (4.4%). The incidence of serious AEs was also low and similar between placebo and mirabegron groups: placebo (2.1%), mirabegron 25 mg (1.6%), mirabegron 50 mg (2.1%), mirabegron 100 mg (2.8%), total mirabegron (2.3%) and tolterodine ER 4 mg (2.2%). Three deaths were reported in this pooled 12-week safety analysis: in the placebo group, a 76-year-old woman died as a result of cardiac arrest 56 days after the last dose of study drug; in the mirabegron 100 mg group, a 66-year-old woman died because of metastatic colon cancer 30 days after the last dose of study drug – neither case was considered to be related to study drug – and in the tolterodine ER 4 mg group, a 74-year-old man died because of a ruptured cerebral aneurysm on day 70 (treatment-emergent), 10 days after the last dose of study drug was administered, and was considered as possibly related to study drug.

In terms of vital sign measurements for blood pressure and pulse rate, the adjusted mean difference vs. placebo for change from baseline to Final Visit in SBP and DBP (AM and PM measurements) was negligible and comparable across each mirabegron group and the tolterodine ER 4 mg group. The adjusted mean difference vs. placebo for change from baseline to Final Visit in pulse rate (AM and PM measurements) increased dose-dependently by 0.6–2.3 bpm in the mirabegron groups and by 1.0–2.1 bpm in the tolterodine ER 4 mg group (Table 4). The overall occurrence of tachycardia events, either based on TEAE and/or observations of pulse rate ≥ 100 bpm captured by patient diary, was less than 5% in each treatment group and comparable with placebo (Table 4). ECG findings revealed no overt trends in central tendency or categorical outliers for QTc interval assessment across treatment groups. Changes in haematology and serum chemistry, including renal parameters, were small and comparable across treatment groups.

**Table 4:** Summary of changes from baseline to Final Visit in blood pressure and pulse rate

	Placebo (*n* = 1380)		Mirabegron 25 mg (*n* = 432)		Mirabegron 50 mg (*n* = 1375)		Mirabegron 100 mg (*n* = 929)		Tolterodine ER 4 mg (*n* = 495)	
					
	AM	PM	AM	PM	AM	PM	AM	PM	AM	PM
**Blood pressure (mmHg)**										
SBP										
*N*	1329	1326	410	410	1327	1327	891	890	476	476
Baseline, mean (SE)	125.9 (0.47)	125.0 (0.41)	129.2 (0.81)	129.0 (0.71)	126.4 (0.47)	125.6 (0.43)	125.0 (0.55)	123.7 (0.49)	128.2 (0.75)	127.4 (0.64)
Final Visit, mean (SE)	126.2 (0.45)	125.7 (0.41)	128.8 (0.75)	128.3 (0.67)	127.2 (0.46)	126.6 (0.41)	125.6 (0.51)	125.2 (0.46)	128.4 (0.70)	127.9 (0.63)
Adjusted change from baseline, mean (SE), (95% CI)†	0.2 (0.25), (−0.3, 0.7)	0.6 (0.25), (0.1, 1.1)	−0.3 (0.52), (−1.3, 0.7)	−0.5 (0.53), (−1.5, 0.6)	0.8 (0.25), (0.3, 1.3)	1.1 (0.25), (0.6, 1.6)	0.6 (0.32), (−0.0, 1.2)	1.4 (0.33), (0.8, 2.1)	0.1 (0.45), (−0.8, 1.0)	0.5 (0.46), (−0.4, 1.4)
Difference vs. placebo, mean (SE), (95% CI)‡	–	–	−0.5 (0.57), (−1.6, 0.6)	−1.0 (0.58), (−2.2, 0.1)	0.6 (0.35), (−0.1, 1.3)	0.5 (0.36), (−0.2, 1.2)	0.4 (0.41), (−0.4, 1.2)	0.9 (0.42), (0.1, 1.7)	−0.1 (0.52), (−1.1, 1.0)	−0.0 (0.53), (−1.1, 1.0)
DBP										
*N*	1329	1326	410	410	1327	1327	890	890	476	476
Baseline, mean (SE)	77.1 (0.26)	75.3 (0.23)	78.2 (0.48)	76.1 (0.46)	77.2 (0.25)	75.4 (0.24)	77.4 (0.30)	75.3 (0.28)	76.8 (0.40)	75.4 (0.38)
Final Visit, mean (SE)	77.2 (0.25)	75.7 (0.24)	77.6 (0.43)	75.7 (0.42)	77.6 (0.24)	76.2 (0.24)	77.7 (0.28)	76.3 (0.28)	77.7 (0.39)	76.6 (0.36)
Adjusted change from baseline, mean (SE), (95% CI)†	0.0 (0.16), (−0.3, 0.3)	0.4 (0.16), (0.1, 0.7)	−0.1 (0.33), (−0.8, 0.5)	0.1 (0.34), (−0.6, 0.7)	0.4 (0.16), (0.1, 0.7)	0.7 (0.16), (0.4, 1.1)	0.2 (0.21), (−0.2, 0.6)	0.9 (0.21), (0.5, 1.3)	0.8 (0.29), (0.2, 1.3)	1.4 (0.30), (0.8, 2.0)
Difference vs. placebo, mean (SE), (95% CI)‡	–	–	−0.1 (0.37), (−0.9, 0.6)	−0.3 (0.37), (−1.0, 0.4)	0.4 (0.22), (−0.1, 0.8)	0.4 (0.23), (−0.1, 0.8)	0.2 (0.26), (−0.3, 0.7)	0.5 (0.27), (−0.0, 1.0)	0.7 (0.33), (0.1, 1.4)	1.0 (0.34), (0.4, 1.7)
**Pulse rate (bpm)**										
*N*	1329	1326	410	410	1327	1327	891	890	476	476
Baseline, mean (SE)	70.5 (0.28)	75.3 (0.29)	71.0 (0.50)	75.5 (0.51)	70.4 (0.28)	74.9 (0.28)	70.4 (0.34)	74.4 (0.34)	69.8 (0.44)	73.9 (0.45)
Final Visit, mean (SE)	70.9 (0.29)	74.7 (0.29)	71.7 (0.52)	75.3 (0.53)	71.8 (0.29)	75.5 (0.28)	72.9 (0.34)	76.5 (0.34)	71.4 (0.44)	76.0 (0.45)
Adjusted change from baseline, mean (SE), (95% CI)†	0.4 (0.17), (0.1, 0.8)	−0.4 (0.18), (−0.8, −0.1)	1.3 (0.36), (0.6, 2.0)	0.2 (0.37), (−0.6, 0.9)	1.4 (0.17), (1.1, 1.8)	0.6 (0.18), (0.2, 0.9)	2.3 (0.22), (1.9, 2.8)	1.9 (0.23), (1.4, 2.3)	1.4 (0.31), (0.8, 2.0)	1.7 (0.32), (1.1, 2.3)
Difference vs. placebo, mean (SE), (95% CI)‡	–	–	0.9 (0.40), (0.1, 1.6)	0.6 (0.41), (−0.2, 1.4)	1.0 (0.24), (0.5, 1.5)	1.0 (0.25), (0.5, 1.5)	1.9 (0.28), (1.3, 2.5)	2.3 (0.29), (1.7, 2.9)	1.0 (0.36), (0.3, 1.7)	2.1 (0.37), (1.4, 2.8)
Incidence of tachycardia (%)*										
Tachycardia events, *N* (%)	43 (3.1)		21 (4.9)		52 (3.8)		43 (4.6)		16 (3.2)	

SBP, systolic blood pressure; DBP, diastolic blood pressure.

* Based on TEAE and/or observations of pulse rate ≥ 100 bpm captured by patient diary.

† Adjusted change from baseline is generated from the ANCOVA model with treatment group, gender and study as fixed factors, and baseline as a covariate.

‡ Differences in the adjusted means are calculated by subtracting the adjusted mean of placebo from the adjusted mean of treatment group.

With respect to other events of interest, the incidence of urinary retention was infrequent in the studies and less in mirabegron-treated patients than in placebo or tolterodine-treated patients: placebo (*n* = 7; 0.5%), mirabegron 25 mg (*n* = 0), mirabegron 50 mg (*n* = 1; 0.1%), mirabegron 100 mg (*n* = 0), total mirabegron (*n* = 1; < 0.1%) and tolterodine (*n* = 3; 0.6%). The mean change from baseline to Final Visit in PVR volume was unremarkable across treatment groups: placebo (−1.6 ml), mirabegron 25 mg (−3.0 ml), mirabegron 50 mg (−0.9 ml), mirabegron 100 mg (−0.7 ml) and tolterodine ER 4 mg (+ 0.1 ml). The proportion of patients experiencing ≥ 150 ml change from baseline in PVR volume was lower in the mirabegron groups compared with placebo and tolterodine: placebo (0.7%), mirabegron 25 mg (0%), mirabegron 50 mg (0.3%), mirabegron 100 mg (0.4%) and tolterodine ER 4 mg (0.8%).

## Discussion

Mirabegron, a β_3_-adrenoceptor agonist, is approved for the treatment of OAB symptoms in Japan, the USA, Canada and Europe, and has a different mechanism of action from the antimuscarinic agents currently available. Mirabegron facilitates detrusor relaxation during the storage phase of the micturition cycle and, in contrast with antimuscarinic agents, improves the storage capacity of the bladder without impairing the amplitude of the contraction during the voiding phase of the micturition cycle 25.

In this pooled analysis of three international phase III studies 21–23, a total of 3542 OAB patients were included in the efficacy analysis, and 4611 OAB patients in the safety analysis, reflective of the general OAB population. More than half of these patients were randomised to mirabegron for 12 weeks at doses of 25, 50 and 100 mg. Furthermore, this pooled dataset included approximately 50% of OAB patients who had been previously treated with antimuscarinic agents but had discontinued either because of insufficient efficacy and/or poor tolerability.

In OAB patients, mirabegron 50 and 100 mg demonstrated significant and clinically meaningful efficacy in terms of improving the symptoms of OAB, including incontinence episodes, micturition frequency and urgency, which are recognised by international guidelines as important outcome measures for OAB therapies 26.

The benefit of mirabegron is evident in both treatment-naïve and previous OAB-treated patients. This is a clinically relevant observation, given the poor persistence rate associated with antimuscarinic drugs – discontinuation rates above 50% and 75% have been reported at 6 months and 1 year, respectively 27,28 – and the fact that, in addition to bothersome AEs (e.g., dry mouth), inadequate response to treatment is an underlying reason for discontinuation of antimuscarinic treatment 27,29–31.

Both 50 and 100 mg doses of mirabegron demonstrated significant improvements compared with placebo for all of the key secondary end-points, including measures of urgency (the core symptom of OAB), and mean volume voided/micturition. Across primary and key secondary end-points, significant improvements with both mirabegron doses were evident at the first assessment period (week 4) and were maintained throughout the 12-week treatment period – an earlier benefit cannot be ruled out but was not assessed prior to the initial 4-week evaluation. Responder analyses showed a significant improvement with mirabegron 50 and 100 mg in terms of dry rates, ≥ 50% reduction in mean number of incontinence episodes/24 h and the proportion of patients with ≤ 8 micturitions/24 h at Final Visit. Taken together, these data demonstrate a clinically meaningful benefit with mirabegron in the objective end-points of OAB.

As OAB is a symptom complex, it can be difficult to determine the efficacy of treatments using objective measures alone. Hence, patient-reported outcomes are increasingly important in the assessment of treatments for symptom-based conditions such as OAB 4. The TS-VAS scores for the 50 and 100 mg mirabegron doses were significantly better and approximately one point higher than patients receiving placebo and reflect the directional parallelism of the subjective and objective improvements in OAB symptoms.

The mirabegron 100 mg dose does not appear to afford a greater efficacy benefit than the 50 mg dose for reduction in incontinence episodes or micturition frequency vs. placebo. A dose–response effect was not evident with the 50 and 100 mg doses across the co-primary and secondary end-points. Although data from the 25 mg mirabegron arm 23 were not included in the pooled efficacy analysis, 25 mg is the recommended starting dose in the USA. In the NCT00912964 phase III study, mirabegron 25 mg showed clinically significant efficacy compared with placebo for the co-primary end-points; however, for the majority of the secondary end-points it did not demonstrate significance vs. placebo 23.

In terms of the safety and tolerability profile of mirabegron, the pooled analysis confirms the results of the individual phase III studies 21–23. The most common AEs (> 3% of total mirabegron group) were hypertension, nasopharyngitis and urinary tract infection. The incidence of hypertension and urinary tract infection decreased as the mirabegron dose increased and both incidences in the total mirabegron group were similar to placebo or tolterodine. The incidence of the most bothersome AE for antimuscarinic drugs, dry mouth, was at placebo level with each mirabegron dose and lower than tolterodine. This finding is consistent with previous randomised, controlled trials of other antimuscarinic therapies, which have reported even higher incidences of dry mouth (9–93%); these studies have also reported high incidences of constipation (4–21%) 32–41. Given that dry mouth is the chief cause of discontinuation because of poor tolerability with antimuscarinic drugs 27,29, the pooled safety data indicate that mirabegron may be a valuable treatment option for patients with OAB.

The pooled safety analysis offers more precise estimates of certain safety parameters, such as vital signs and AEs related to cardiovascular safety, than the individual phase III studies. In this pooled analysis, mirabegron was associated with approximate increases of ≤ 1 mmHg in blood pressure and ≥ 1 bpm in pulse rate vs. placebo. The incidence of hypertension was similar among the total mirabegron group, placebo and tolterodine. The incidence of urinary retention was greater in the placebo and tolterodine groups than in mirabegron-treated patients. ECG, PVR volume and laboratory data were unremarkable across each treatment group. Overall, the data support the good safety profile of mirabegron based on this pooled phase III clinical study dataset.

## Conclusions

Mirabegron, a β_3_-adrenoceptor agonist, and the first in its class for the treatment of OAB symptoms, has a distinct mechanism of action from antimuscarinic agents, the current pharmacological mainstay in treating OAB. In these pooled analyses of efficacy and safety, mirabegron administered at daily doses of 50 and 100 mg for 12 weeks demonstrated significant and clinically meaningful improvement in treating the symptoms of OAB, including urinary incontinence, micturition frequency and urgency. Mirabegron was beneficial in both treatment-naïve patients and in patients who used prior antimuscarinic treatment. The improvement in OAB symptoms measured objectively was reflected in the improvement in the patient-reported outcome related to treatment satisfaction.

Mirabegron was well tolerated with an incidence of dry mouth at placebo level and lower than tolterodine. Mirabegron represents a new therapeutic in a new class of medication for the treatment of OAB and has demonstrable efficacy and tolerability in a large clinical trial dataset in OAB patients. In clinical practice, mirabegron will provide a valuable treatment option for OAB syndrome.

## References

[1] Abrams P, Cardozo L, Fall M (2003). The standardisation of terminology in lower urinary tract function: report from the standardisation sub-committee of the International Continence Society. Urology.

[2] Wein AJ, Rovner ES (1999). The overactive bladder: an overview for primary care health providers. Int J Fertil Womens Med.

[3] Coyne KS, Sexton CC, Thompson CL (2009). The prevalence of lower urinary tract symptoms (LUTS) in the USA, the UK and Sweden: results from the Epidemiology of LUTS (EpiLUTS) study. BJU Int.

[4] Brubaker L, Chapple C, Coyne KS, Kopp Z (2006). Patient-reported outcomes in overactive bladder: importance for determining clinical effectiveness of treatment. Urology.

[5] Coyne KS, Sexton CC, Irwin DE, Kopp ZS, Kelleher CJ, Milsom I (2008). The impact of overactive bladder, incontinence and other lower urinary tract symptoms on quality of life, work productivity, sexuality and emotional well-being in men and women: results from the EPIC study. BJU Int.

[6] Homma Y, Yamaguchi O, Hayashi K, Committee NBS (2005). An epidemiological survey of overactive bladder symptoms in Japan. BJU Int.

[7] Milsom I, Abrams P, Cardozo L, Roberts RG, Thüroff J, Wein AJ (2001). How widespread are the symptoms of an overactive bladder and how are they managed? A population-based prevalence study. BJU Int.

[8] Stewart WF, Van Rooyen JB, Cundiff GW (2003). Prevalence and burden of overactive bladder in the United States. World J Urol.

[9] MacDiarmid SA (2008). Maximizing the treatment of overactive bladder in the elderly. Rev Urol.

[10] Michel MC, Chapple CR (2009). Basic mechanisms of urgency: preclinical and clinical evidence. Eur Urol.

[11] Steers WD (2002). Pathophysiology of overactive bladder and urge urinary incontinence. Rev Urol.

[12] Yamaguchi O, Chapple CR (2007). β_3_-adrenoceptors in urinary bladder. Neurourol Urodyn.

[13] Igawa Y, Yamazaki Y, Takeda H (1999). Functional and molecular biological evidence for a possible β_3_-adrenoceptor in the human detrusor muscle. Br J Pharmacol.

[14] Takeda M, Obara K, Mizusawa T (1999). Evidence for β_3_-adrenoceptor subtypes in relaxation of the human urinary bladder detrusor: analysis by molecular biological and pharmacological methods. J Pharmacol Exp Ther.

[15] Wuest M, Eichhorn B, Grimm MO, Wirth MP, Ravens U, Kaumann AJ (2009). Catecholamines relax detrusor through beta 2-adrenoceptors in mouse and beta 3-adrenoceptors in man. J Pharmacol Exp Ther.

[16] Yamanishi T, Chapple CR, Yasuda K, Yoshida K, Chess-Williams R (2003). Role of β-adrenoceptor subtypes in mediating relaxation of the pig bladder trigonal muscle in vitro. Neurourol Urodyn.

[17] Andersson KE (2009). Prospective pharmacologic therapies for the overactive bladder. Ther Adv Urol.

[18] Leon LA, Hoffman BE, Gardner SD (2008). Effects of the β_3_-adrenergic receptor agonist disodium 5-[(2R)-2-[[(2R)-2-(3-chlorophenyl)-2-hydroxyethyl]amino]propyl]-1,3-benzodioxole-2,2-dicarboxylate (CL-316243) on bladder micturition reflex in spontaneously hypertensive rats. J Pharmacol Exp Ther.

[19] Tyagi P, Tyagi V (2010). Mirabegron, a β_3_-adrenoceptor agonist for the potential treatment of urinary frequency, urinary incontinence or urgency associated with overactive bladder. IDrugs.

[20] Takasu T, Ukai M, Sato S (2007). Effect of (R)-2-(2-aminothiazol-4-yl)-4′-{2-[(2-hydroxy-2-phenylethyl)amino]ethyl} acetanilide (YM178), a novel selective β_3_-adrenoceptor agonist, on bladder function. J Pharmacol Exp Ther.

[21] Nitti V, Auerbach S, Martin N, Calhoun A, Lee M, Herschorn S (2013). Results of a randomized phase III trial of mirabegron in patients with overactive bladder. J Urol.

[22] Khullar V, Amarenco G, Angulo JC (2013). Efficacy and tolerability of mirabegron, a β_3_-adrenoceptor agonist, in patients with overactive bladder: results from a randomised European-Australian phase 3 trial. Eur Urol.

[23] Herschorn S, Barkin J, Castro-Diaz D (2013). A phase III, randomized, double-blind, parallel-group, placebo-controlled, multicentre study to assess the efficacy and safety of the beta-3 adrenoceptor agonist, mirabegron in patients with symptoms of overactive bladder. Urology.

[24] Cartwright R, Srikrishna S, Cardozo L, Robinson D (2011). Validity and reliability of the patient's perception of intensity of urgency scale in overactive bladder. BJU Int.

[25] Frazier EP, Peters SL, Braverman AS, Ruggieri MR, Michel MC (2008). Signal transduction underlying the control of urinary bladder smooth muscle tone by muscarinic receptors and beta-adrenoceptors. Naunyn Schmiedebergs Arch Pharmacol.

[26] Lucas MG, Bosch JLHR, Cruz FR http://www.uroweb.org/gls/pdf/18_Urinary_Incontinence_LR.pdf.

[27] Benner JS, Nichol MB, Rovner ES (2010). Patient-reported reasons for discontinuing overactive bladder medication. BJU Int.

[28] Brostrom S, Hallas J (2009). Persistence of antimuscarinic drug use. Eur J Clin Pharmacol.

[29] D'Souza AO, Smith MJ, Miller LA, Doyle J, Ariely R (2008). Persistence, adherence, and switch rates among extended-release and immediate-release overactive bladder medications in a regional managed care plan. J Manag Care Pharm.

[30] Shaya FT, Blume S, Gu A, Zyczynski T, Jumadilova Z (2005). Persistence with overactive bladder pharmacotherapy in a Medicaid population. Am J Manag Care.

[31] Wagg A, Compion G, Fahey A, Siddiqui E (2012). Persistence with prescribed antimuscarinic therapy for overactive bladder: a UK experience. BJU Int.

[32] Appell RA, Sand P, Dmochowski R (2001). Prospective randomized controlled trial of extended-release oxybutynin chloride and tolterodine tartrate in the treatment of overactive bladder: results of the OBJECT study. Mayo Clin Proc.

[33] Cardozo L, Hessdörfer E, Milani R (2008). Solifenacin in the treatment of urgency and other symptoms of overactive bladder: results from a randomized, double-blind, placebo-controlled, rising-dose trial. BJU Int.

[34] Chapple CR, Rechberger T, Al-Shukri S (2004). Randomized, double-blind placebo- and tolterodine-controlled trial of the once-daily antimuscarinic agent solifenacin in patients with symptomatic overactive bladder. BJU Int.

[35] Diokno AC, Appell RA, Sand PK (2003). Prospective, randomized, double-blind study of the efficacy and tolerability of the extended-release formulations of oxybutynin and tolterodine for overactive bladder: results of the OPERA trial. Mayo Clin Proc.

[36] Schaefer W (2009). Solifenacin in the treatment of urgency and other symptoms of overactive bladder: results from a randomized, double-blind, placebo-controlled, rising-dose trial. BJU Int.

[37] Staskin D, Sand P, Zinner N, Dmochowski R (2007). Once daily trospium chloride is effective and well tolerated for the treatment of overactive bladder: results from a multicenter phase III trial. J Urol.

[38] Staskin DR, Rosenberg MT, Sand PK, Zinner NR, Dmochowski RR (2009). Trospium chloride once-daily extended release is effective and well tolerated for the treatment of overactive bladder syndrome: an integrated analysis of two randomised, phase III trials. Int J Clin Pract.

[39] Thuroff JW, Chartier-Kastler E, Corcus J (1998). Medical treatment and medical side effects in urinary incontinence in the elderly. World J Urol.

[40] Zinner N, Kobashi KC, Ebinger U (2008). Darifenacin treatment for overactive bladder in patients who expressed dissatisfaction with prior extended-release antimuscarinic therapy. Int J Clin Pract.

[41] Zinner N, Tuttle J, Marks L (2005). Efficacy and tolerability of darifenacin, a muscarinic M3 selective receptor antagonist (M3 SRA), compared with oxybutynin in the treatment of patients with overactive bladder. World J Urol.

